# Fetal environment

**DOI:** 10.4103/0971-3026.43848

**Published:** 2008-11

**Authors:** Arun Kinare

**Affiliations:** Department of Ultrasound, K.E.M. Hospital, Jehangir Hospital, Pune, India

**Keywords:** Amniotic fluid, placenta, ultrasound (USG), umbilical cord

## Abstract

The intrauterine environment has a strong influence on pregnancy outcome. The placenta and the umbilical cord together form the main supply line of the fetus. Amniotic fluid also serves important functions. These three main components decide whether there will be an uneventful pregnancy and the successful birth of a healthy baby. An insult to the intrauterine environment has an impact on the programming of the fetus, which can become evident in later life, mainly in the form of cardiovascular diseases, diabetes, and certain learning disabilities. The past two decades have witnessed major contributions from researchers in this field, who have included ultrasonologists, epidemiologists, neonatologists, and pediatricians. Besides being responsible for these delayed postnatal effects, abnormalities of the placenta, umbilical cord, and amniotic fluid also have associations with structural and chromosomal disorders. Population and race also influence pregnancy outcomes to some extent in certain situations. USG is the most sensitive imaging tool currently available for evaluation of these factors and can offer considerable information in this area. This article aims at reviewing the USG-related developments in this area and the anatomy, physiology, and various pathologies of the placenta, umbilical cord, and the amniotic fluid.

## Placental development

Proliferation of trophoblastic tissue takes place at a rapid pace in the early stages of gestation. Formation of villi begins at around the 13^th^ day after conception. The villi project into the intervillous spaces. As they grow towards the decidua, the villi atrophy to form the chorion frondosum. The intervillous spaces contain a large blood sinus. The intervillous flow is recognizable only after the 12^th^ week of gestation; this is the time when the trophoblastic plugs are dislocated and a fully established maternal circulation becomes appreciable.[1]

**Figure 1:**
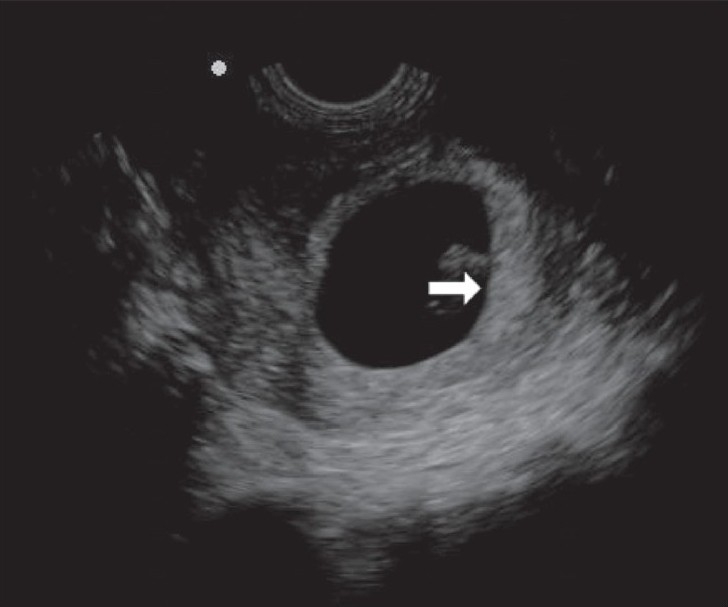
Implantation site of placenta; 8-weeks' sac. A hyperechoic focus can predict the implantation site (arrow)

**Figure 2:**
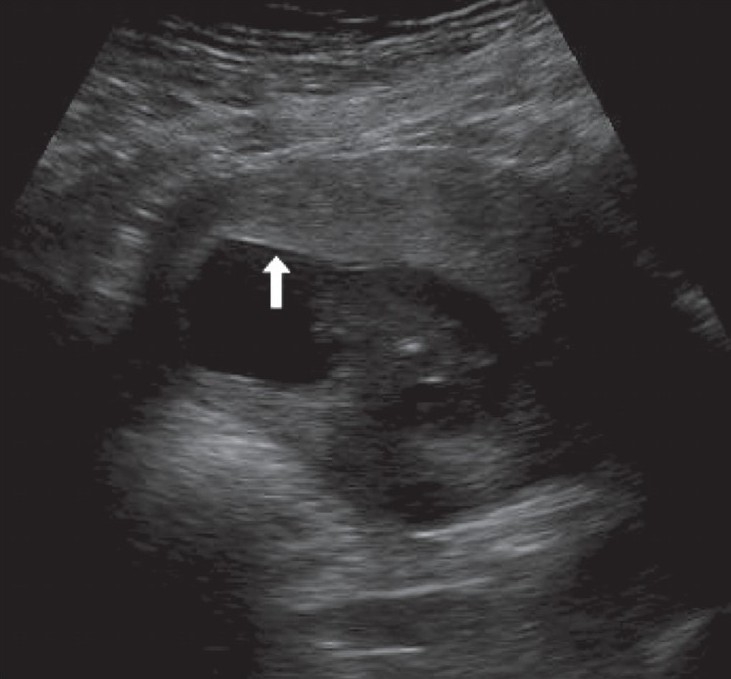
Placenta. Reliable confirmation of location is seen at 11 weeks; in this case an anterior placenta (arrow)

The implantation site of the placenta can be identified on sonography as a hyperechoic focus in the early period of gestation [Figure 1]. The site of implantation is known to have an association with perinatal outcome. Lateral placentation is seen in fetuses with intrauterine growth restriction (IUGR).[2] Implantation occurs at around 8–10 weeks, when the placenta is formed by the interaction of the deciduas basalis of the endometrium and the chorionic villi of the fetus. True definition of the placenta is possible only 10–11 weeks after conception [Figure 2].

## Placental size

Several researchers have studied placental thickness, volume, and diameter and established the relationships of these factors with the birth outcome. The placental size is principally determined in the first half of pregnancy and the bulk of the volume is achieved by mid-pregnancy.[3] Barker's group attaches a lot of importance to placental size as a predictor of hypertension in middle age.[4] While placental thickness and diameter can be measured easily, volume estimation requires special techniques.

## Placental thickness

Placental thickness is measured at its midportion, from the chorionic plate to the basilar plate, on a longitudinal plane. It should exclude any abnormalities such as fibroids, myometrial contractions, or venous lakes. By and large, the placental thickness in millimeters corresponds to the weeks of gestation. Rarely, placental thickness can exceed 4 cm at term.[5]

## Placental diameter

As with placental thickness, the estimation of placental diameter is quite simple. The diameter is measured in the transverse section by calculating the maximum dimensions of the chorionic surface.

## Placental volume

In contrast to the measurement of thickness and diameter, the method for placental volume measurement is rather complicated. Different methods have been used by different people. Initial attempts at measuring the placental volume made use of static methods. Though real-time scanning was used by some authors, the first reliable method was developed by Howe *et al.*[6] This was a parallel planimetric method using a special software and real-time USG. A large-footprint linear-array transducer is a requirement for this method.

Recently, three-dimensional (3D) USG has been used for placental volume measurement. The preferred time of measurement is at the end of the first trimester.[7] Placental volume seems to vary with the population under study and the results of the only Indian study available support this.[8] The median placental volume in our study group was 144 ml as against 292 ml in David Howe's study group. A diminished placental size is a good predictor of IUGR.[9]

Maternal race and a history of use of alcohol, smoking, and hypertension should be taken into consideration when commenting on the placental size.[10] On the other hand, a sonographically thick placenta (placentomegaly) is also considered a risk factor for poor perinatal outcome, primarily due to coexisting fetal anomalies; it is also associated with small-for-gestational-age as well as large-for-gestational-age neonates.[11]

Placentomegaly has a known association with diabetes, intrauterine infections [commonly cytomegalovirus (CMV) infection], chromosomal anomalies, nonimmune hydrops, maternal and fetal anemia, etc. Though the pregnancy outcome can be normal in a case of placentomegaly, it is necessary to have a more careful look at the fetus, especially after intrauterine infections, diabetes, and IUGR have been excluded. The index of suspicion for an anomaly should be high when the placental size is more than the 90^th^ percentile. A history of placentomegaly may be familial in certain rare conditions.[12] Placental mesenchymal dysplasia is a relatively recently recognized entity and is basically a vascular abnormality of the placenta. The USG and histopathological features of this condition simulate a mole. The placentomegaly in this condition can have a normal pregnancy outcome though, in extreme cases, IUGR and evidence of the Beckwith–Wiedmann syndrome have been described.[13]

Placentomegaly in cytomegalovirus infection has implications for the perinatal outcome, as the virus infects the placenta first; fetal disease can be significantly reduced by treating with CMV hyperimmune globulin. Thus, in this case, a thickened placenta can be considered a nonspecific marker of fetal disease.[14]

A relatively smaller placental volume in early gestation has been reported in cases of trisomy 13 and 18. However, there is no strong association of placental volume with chromosomal defects.[15] Poor placental growth in early pregnancy may contribute to low birth weight.[8]

The normal placenta is discoid in shape. Abnormal placental shapes include circumvallate placenta, either complete or partial, and succenturiate lobe. The complications associated with these abnormal shapes can be serious and the list includes placental abruption, preterm delivery, perinatal death, and IUGR (associated with circumvallate placenta). With a succenturiate lobe, there is a higher incidence of velamentous insertion of the cord and vasa previa, as also retention of the placenta.[16]

Placental grading has no importance in the current scenario and should not be included in the routine protocol unless one comes across a grade 3 placenta in the early stages of gestation.

## Placental masses

**Figure 3 (A-B):**
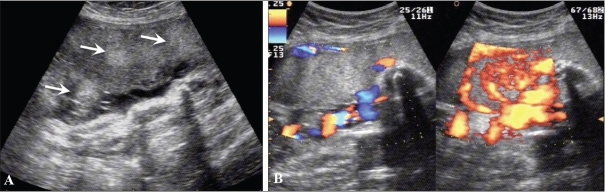
25-weeks' gestation with a rather bulky placenta. Note the focal hyperechoic areas (arrows in A) in an anteriorly located placenta. Color Doppler is not of great help but power Doppler (B) demonstrates the abnormal vascularity in this chorioangioma (image on the right)

Amongst the placental masses, placental chorioangiomas are the most common benign tumors and are seen in approximately 1% of pregnancies. The tumors are usually hypoechoic and may show the presence of calcifications. Chorioangioma can simulate a myoma, a large venous lake, placental teratoma, and incomplete mole. Though these tumors can go unnoticed throughout gestation, they are known to cause complications like polyhydramnios, premature delivery, fetal demise, IUGR, and nonimmune hydrops. Structural anomalies in the fetus are a known association. On B-mode imaging, the tumors can go unnoticed until a late stage of gestation. The Doppler findings are also known to be deceptive, and the tumors can be vascular or avascular [Figure 3]. It is known that a tumor that was vascular in the early stage of gestation can become avascular at a later stage of gestation. A normal outcome of the pregnancy associated with chorioangiomas is also very well known. In general, tumors more than 5 cm in size have a poor pregnancy outcome.[17] Some authors find color Doppler more useful; they feel that the tumors have abundant vascularity and find color Doppler useful in differentiating the tumor from hematomas.[18]

**Figure 4 (A-B):**
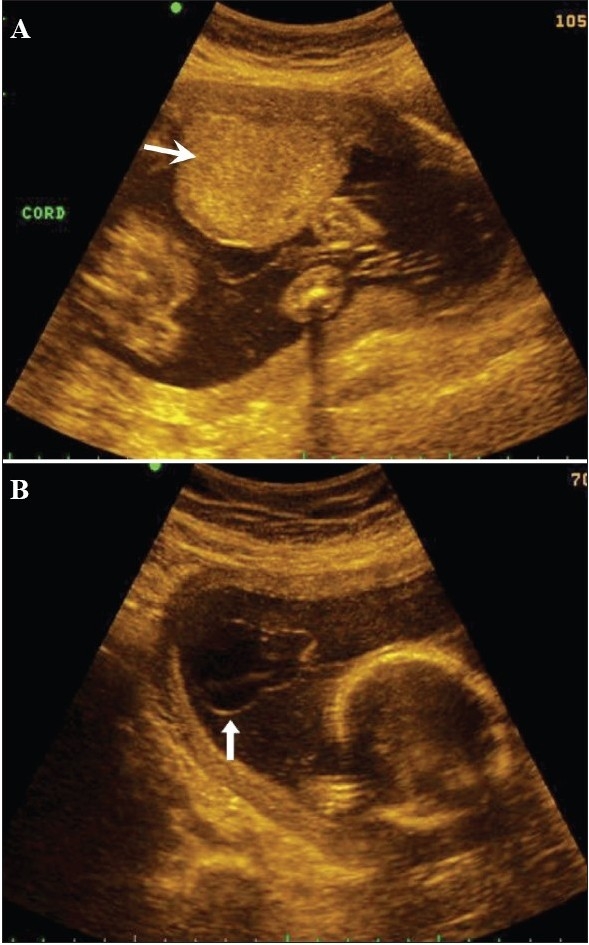
A case of significant IUGR detected at 22 weeks. A small but globular anterior placenta (arrow in A) is seen with multiple subamniotic cysts (arrow in B). Multiple anomalies were confirmed at termination)

**Figure 5 (A, B):**
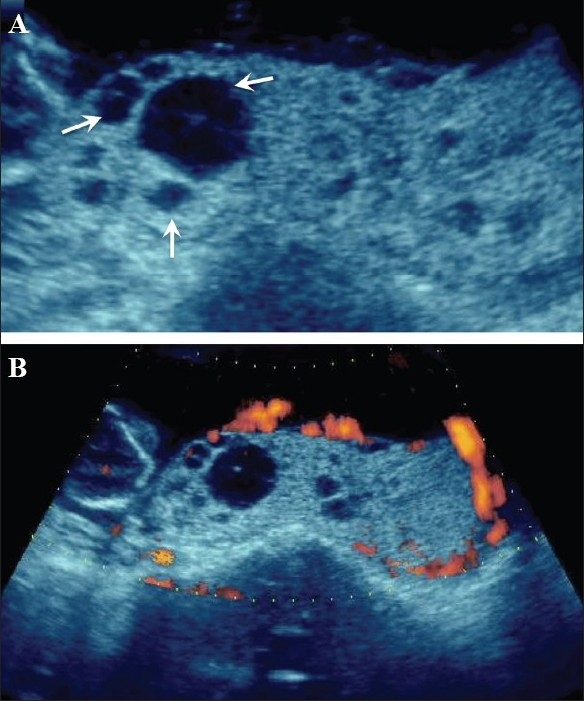
Placental lakes. Cystic foci (arrows in A) are seen in the placenta. Color Doppler (B) helps to exclude molar changes or a tumor

Placental cysts have a controversial etiology but, in general, the outcome of the pregnancy is normal. They are known by different names, such as chorionic, subchorionic, subamniotic, or membranous cysts [Figure 4]. Only large cysts may have an association with IUGR.[19] Placental cysts may simulate venous lakes [Figure 5]. At increased gain settings one may visualize the flow in the venous lakes.

## Hemorrhage related to the placenta

**Figure 6 (A, B):**
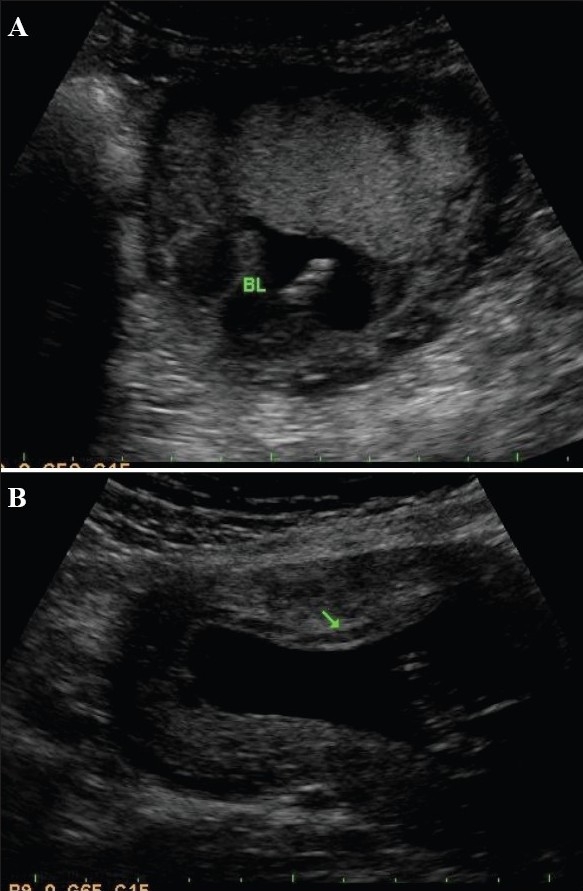
Subchorionic bleeds in two different patients. A small bleed (BL) is seen related to the fundal end of the placenta (A). A small bleed is seen involving most of the anterior placenta (arrow in B). Both bleeds are somewhat old as they are quite echofree in nature

A marginal subchorionic hematoma is the commonest situation in the early period of gestation [Figure 6]. USG appearances vary with the duration of gestation. When small, in very early gestation, they are physiological. They can also mimic a twin sac.[20]

## Placental abruption

Placental abruption is defined as the premature separation of the normally implanted placenta. The condition can be very threatening to the fetus as well as the mother. History is very important and hypertension, advanced maternal age, diabetes, cigarette smoking, and a history of previous abruption are relevant factors. It should be remembered that the diagnosis is purely clinical. The role of USG is to follow the conservatively managed pregnancies.

**Figure 7:**
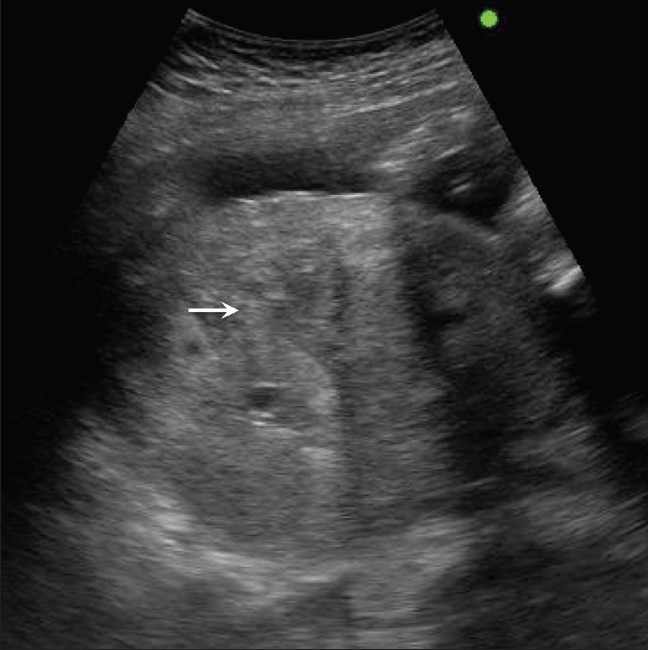
Acute placental abruption. Note the bulky heterogenous placenta (arrows) in this hypertensive, 29-weeks pregnant patient

**Figure 8 (A, B):**
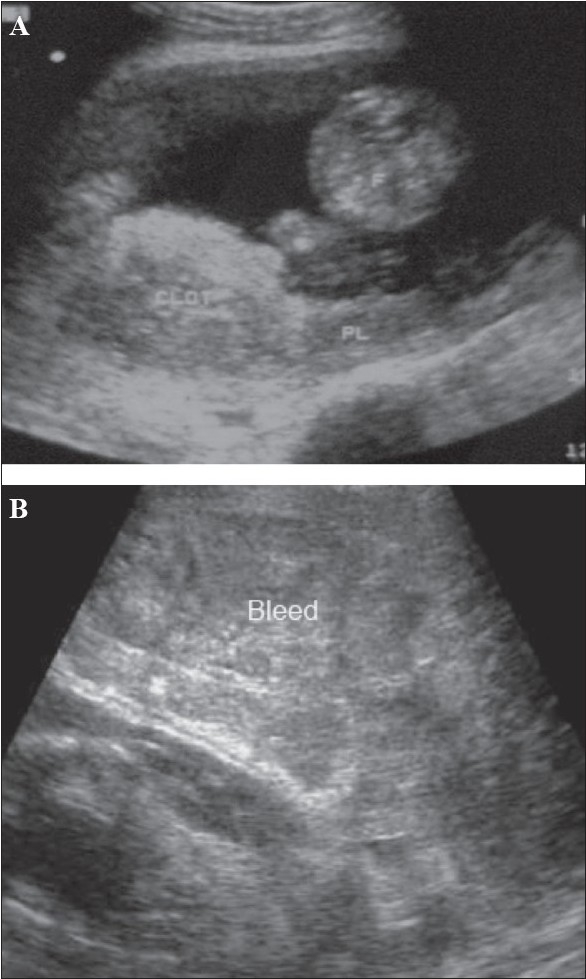
Placental abruption in two different patients. Sagittal section (A) shows an echogenic bleed (CLOT) close to the fetal surface of the placenta in this case of abruption. The bleed is relatively fresh. (B) Another case of acute placental abruption, in which USG shows a few small hypoechoic areas of bleed (Bleed) in the retroplacental bed

It is not uncommon to have a normal USG picture in the presence of a frank abruption. Positive USG features of abruption include a bulky or an enlarged, globular placenta[21] and the presence of a retroplacental or retromembranous clot [Figures 7 and 8]. Follow-up is done with the aim of monitoring the clot size and the USG features of the clot. An organized clot is more echogenic. As time passes, the clots become echo-free. Hematomas can exist at different locations. Subchorionic hematomas are more common prior to 20 weeks, whereas after 20 weeks, retroplacental hematomas are encountered more often. Preplacental hematomas are the least common.[22] Abruptions can be confused with myometrial contraction and myomas.

## Placenta previa

**Figure 9:**
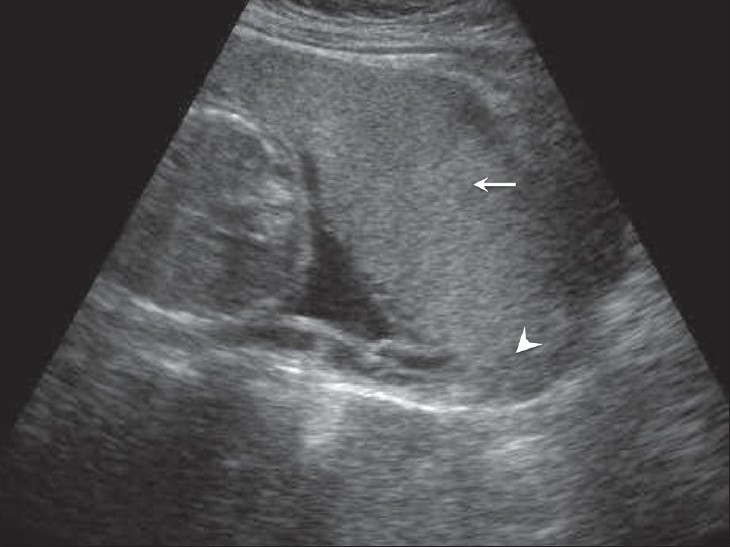
Anterior placenta previa. The placenta (arrow) has covered the internal os completely (arrowhead)

A placenta previa is a placenta that is in front of or ‘previous’ to the birth canal; it remains the primary cause of third trimester bleeding. The incidence of placenta previa is more in multiparous women with a history of Cesarean section. Broadly, two types of previa are recorded: complete and incomplete. In the former, the os is completely covered by the placenta [Figure 9], whereas in the latter the placenta either partially covers or just reaches up to the os. A low-lying placenta is one whose edge is within 2 cm of the os. These low-lying placentae usually migrate. Placental migration is a loose term as the placenta does not itself move; instead, as the uterus expands, the placenta appears to migrate. Low-lying or incomplete placenta previas are common in the second trimester but only a minority persist into the late third trimester. Translabial and transvaginal USG are eminently useful for the detection of placenta previa. Translabial USG has a greater advantage in a posteriorly located placenta. Posteriorly implanted placentae are less likely to migrate as compared to anterior placentae.[23] A large clot from abruption may simulate placenta previa and color mapping can play an important role in differentiating between the two conditions. It is mandatory to have an empty bladder before performing a scan for suspected placenta previa. Safety issues should not preclude the use of the transvaginal route for the diagnosis of previa as the procedure is very safe.[24]

**Figure 10:**
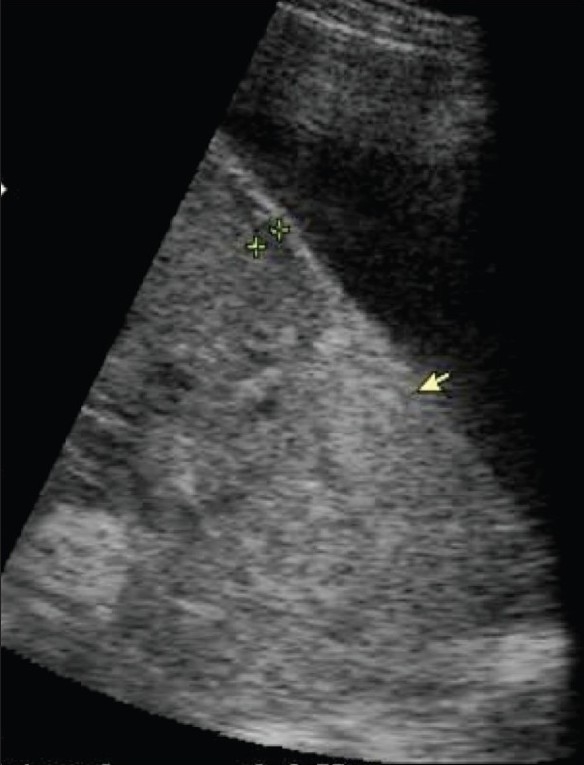
Placenta accreta. The arrow indicates loss of definition of the normal hypoechoic myometrium. The uterine and bladder wall demarcation is also poor

Women with placenta previa are at an increased risk of having a morbidly adherent placenta, especially when there has been a short Cesarean-to-conception interval. Abnormal adherence of the placenta to the uterus beyond the endometrium, with subsequent failure of the placenta to separate even after delivery of the fetus, is termed as placenta accreta. Incidence of accrete is about 1 in 5000 pregnancies. USG diagnostic criteria include loss of the normal hypoechoic myometrium, disruption of the normal uterine serosa–bladder interface, and presence of focal exophytic masses [Figure 10].[25] Accreta vera is the term used when the placenta does not invade the myometrium. In placenta increta, the villi invade the myometrium, whereas in placenta percreta the villi penetrate beyond the uterus, often into the bladder and the rectum. Doppler imaging can identify suspected placenta accreta.[26] In doubtful cases, MRI plays an important role and the use of this modality needs to be explored further.[27]

## Vasa previa

**Figure 11:**
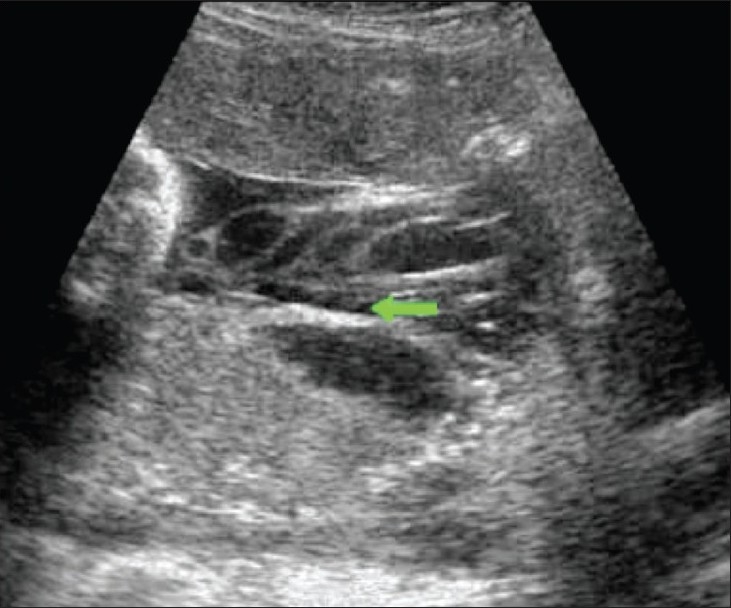
Velamentous cord insertion. The cord insertion is seen along the membranes (arrow). Extension of these splayed vessels into the cervix is not seen in this illustration (Image courtesy Dr Manoj Chinchwadkar)

The incidence of vasa previa varies. In this condition there is velamentous cord insertion across the cervix [Figure 11]. High perinatal mortality is associated with this condition and therefore diagnosis is important. Color flow mapping done transvaginally or translabially should clinch the diagnosis. Flash artifact can mimic a vessel near the os and be responsible for a false positive diagnosis. The artifact, however, is transient, and repeating the examination can confirm the diagnosis.[28]

## Mole

**Figure 12:**
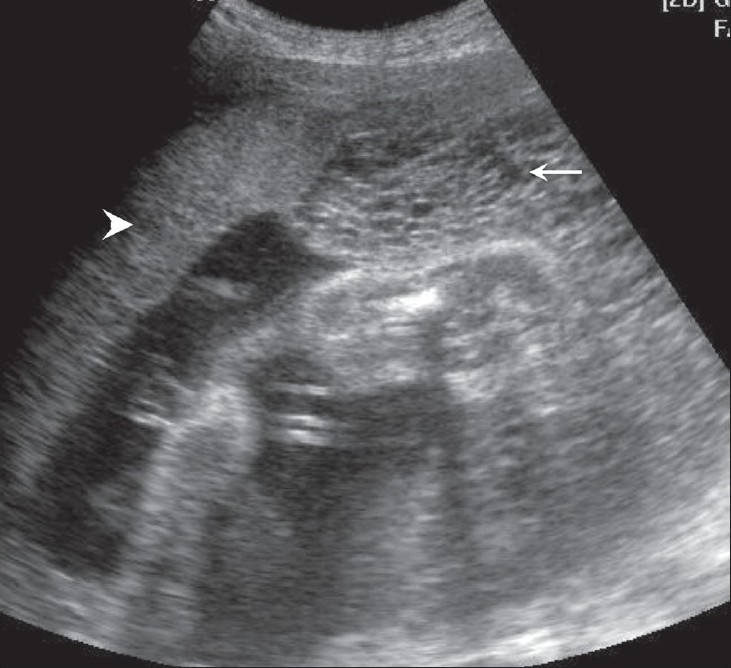
Twin gestation. Molar changes are seen in the first placenta. Sagittal image shows both placentae to be anterior. A typical bunch-ofgrapes appearance (arrow) is present in the first placenta. The second placenta (arrowhead) is normal

**Figure 13 (A,B):**
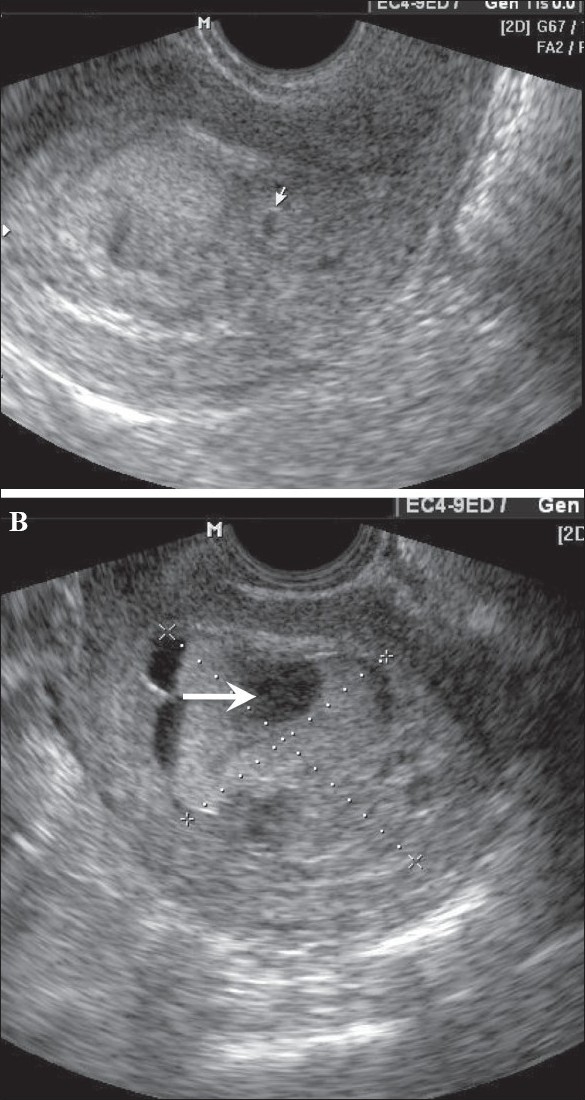
Hydatidiform mole. In this patient with a 6-weeks' gestation presenting with vaginal bleeding, transvaginal USG (A) shows a gestational sac (white arrow) on the first examination. Follow-up examination after 2 days (B) shows focal cystic changes (arrow) with loss of normal definition of the gestational sac, suggesting the possibility of molar changes. Investigations confirmed triploidy

**Figure 14 (A–C):**
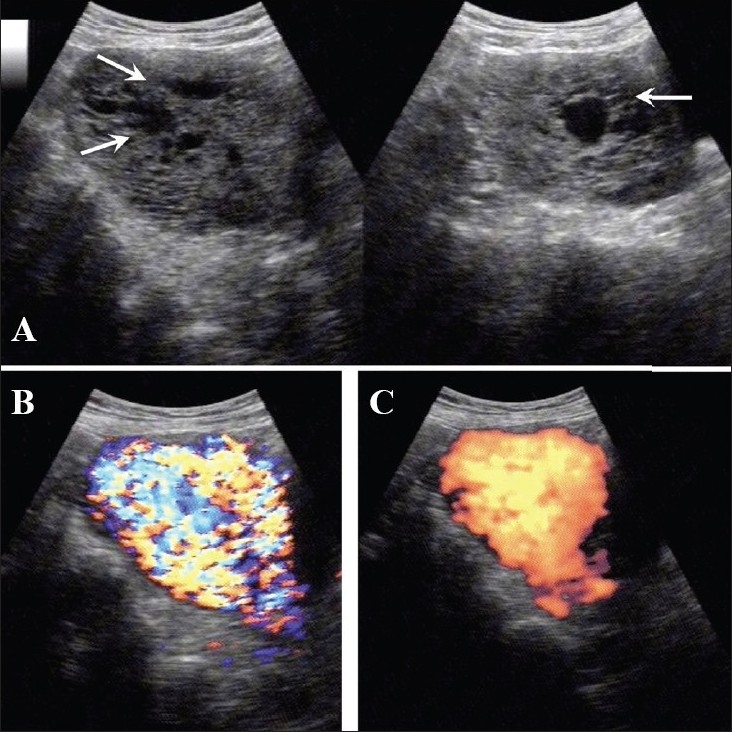
(A) Invasive mole. Extensive cystic foci (arrows in A) are seen in the uterine cavity; the lesion also shows intense vascularity on color Doppler (B) and almost complete filling of the uterus with color on power Doppler (C)

Hydatidiform mole falls in the general category of gestational trophoblastic disease. The group includes molar pregnancy, invasive mole, and choriocarcinoma. Complete and partial moles are the two types of molar pregnancies. The two conditions are differentiated histopathologically. USG appearances of a mole are quite typical, though the classical bunch-of-grapes or snowstorm appearance may not be seen always [Figure 12]. On USG, it is seen as a complex and echogenic intrauterine mass, containing small cystic spaces, which correspond to the hydropic villi. In case the villi are too small, an echogenic mass may be the only feature seen [Figure 13]. It can also be seen as a large fluid collection in a cavity, in which case it may simulate the appearance of retained products of an abortion. A partial mole has a triploid or a tetraploid karyotype and a hydropic placenta is always evident.[29] Color and power mode are usually confirmatory and the classical ‘ball of vascularity’ is encountered in the majority of cases. USG also plays a key role in monitoring the response to therapy in treated cases and, along with β-human chorionic gonadotropin (HCG) estimation, forms the backbone of the approach to treatment. Carter *et al.* found the uterine artery pulsatility index a strong prognostic parameter; it also correlated well with the β-HCG titers. In the first and early second trimester, the flow is typically high velocity and low impedance, which is contrary to what is seen in normal gestation. Color Doppler is also extremely useful in the assessment of invasive disease [Figure 14]. Vascular cystic foci can be reliably identified in the myometrium in these cases.[30]

## Placental infarction

**Figure 15 (A,B):**
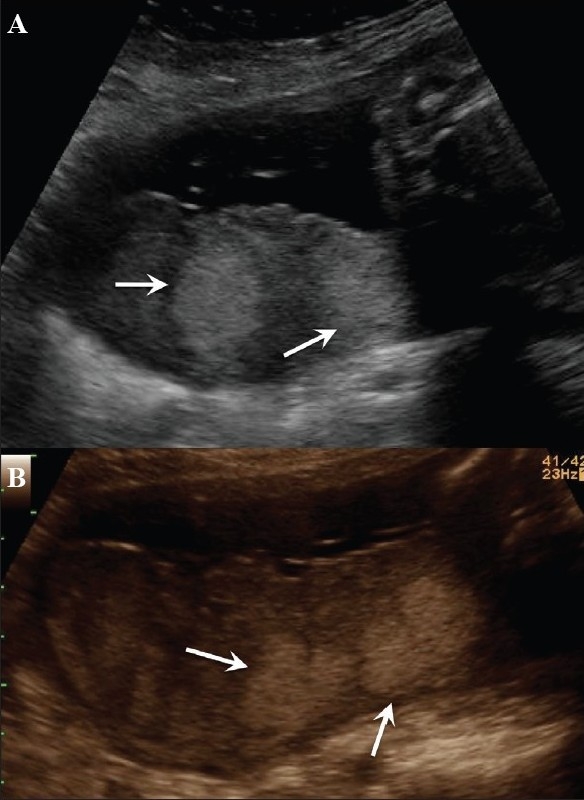
(A) Posterior placenta. The echodense foci (arrows in A) in this case of pregnancy-induced hypertension with IUGR were considered to be likely infarcts, better defined on the chroma image (arrows in B)

**Figure 16:**
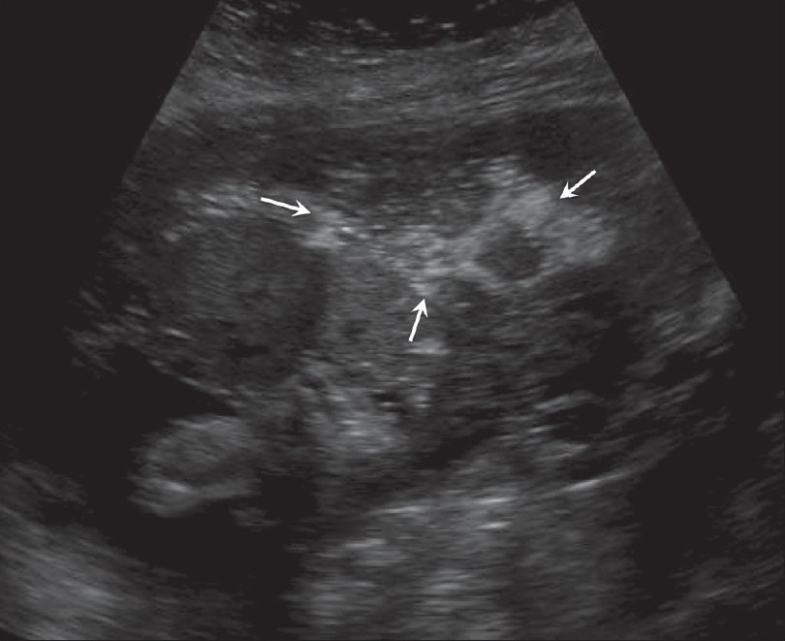
Fibrin deposits. Echo-rich foci (arrows) in the placenta are a normal finding and the peripheral location is typical. These should not be confused with calcifications

Placental infarction is predominantly a histopathological diagnosis. Placental infarcts are only infrequently found on USG. However, with high-resolution equipment one can predict its presence by demonstrating ill-defined hyperechoic areas in the placenta [Figure 15]. The presence of IUGR, pregnancy-induced hypertension, and post-term pregnancy increases the likelihood of infarcts. Small infarcts are often encountered in normal pregnancies but they do not have any adverse effect. Very often the interpretation of infarction is subjective. Fibrin deposition in the placenta is not a true infarct and usually is of no clinical importance [Figure 16]. The role of Doppler in cases of infarcts is questionable. However, demonstration of intraplacental vasculature can be of interest. Reports in the literature suggest that fetal distress can be predicted when the intraplacental vasculature is not detectable on color Doppler.[31]

## Umbilical cord

The umbilical cord, the main component of the fetal supply line, is undoubtedly the most neglected structure in routine USG evaluation of pregnancy. The importance of cord assessment is underestimated and, as a result, cord abnormalities are underreported. The cord is more like an organ and can have various designs. The growth, development, and the structure of the umbilical cord depend upon various factors and any insult to these, results in serious fetal compromise. The cord deserves attention right from the first trimester. The probability of identifying congenital anomalies will be much higher if careful cord assessment is attempted in the earlier periods of gestation.

## Development and anatomy of the umbilical cord

**Figure 17:**
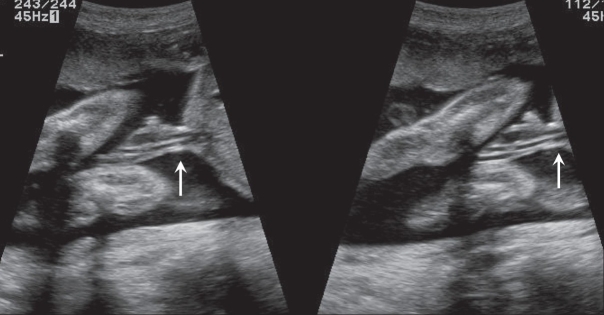
Normal umbilical cord – fetal insertion (arrows). This is the preferred area for morphometry

**Figure 18:**
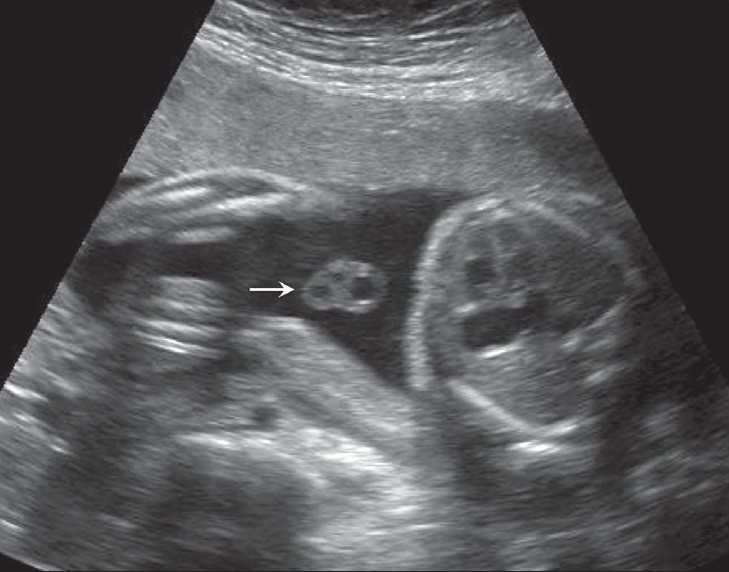
A transverse section of the cord (arrow) is the best to show the number of vessels

The umbilical cord is formed in the early period of embryogenesis from the body stalk (umbilical arteries) vein and the allantois and the yolk stalk (omphalomesenteric stalk and the remnant of the original yolk sac attachment). The cord is first visualized at around the 8^th^ week, when its length is approximately equal to the crown–rump length (CRL). At this stage, the cord is a rather thick structure. The growth of the cord continues till about 36 weeks, though most of the growth is complete by 28 weeks. Wharton's jelly acts like a cushion around the cord. Its elasticity and the cushion-like effect protect the cord from bending, vibrations, and stretching by an active fetus. The cord has two arteries and one vein [Figures 17 and 18]; their relationship can vary and various normal structural patterns are observed. The total cord area and the Wharton's jelly area both decrease at near term. The average length of the cord at term is 61 cm. Cords less than 32 cm are termed short cords. The cord is shorter in twins than in singletons. Pulsations of the cord occur at the same rate as the fetal heart rate and may be appreciable during real-time USG evaluation. The placental insertion of the cord may be central or eccentric. An exaggerated form of eccentric placental insertion is the battledore placenta, where the insertion is at the margin of the placenta; however, this has no clinical significance.[32]

**Figure 19:**
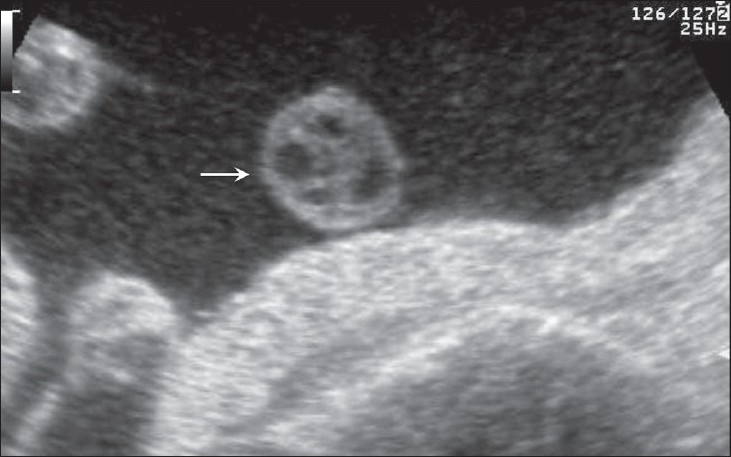
A tranverse section of the cord (arrow) shows a four-vessel cord

Variations in the number of cord vessels are known, the commonest variant form being a single umbilical artery. The presence of any variation warrants a careful search for other anomalies. Four-vessel and five-vessel cords have been described [Figure 19] and the incidence of anomalies in these pregnancies is higher. Fused umbilical arteries are known. Hypoechoic and hyperechoic cords are also known to be associated with a poor pregnancy outcome.[32]

**Figure 20:**
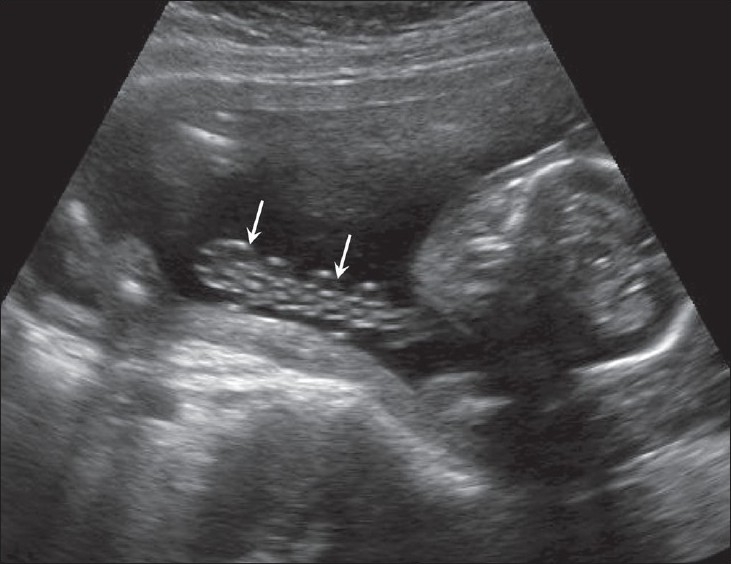
Coiled cord loops (arrows) are well-visualized

Other variations in the cord morphology include altered relationships of artery and vein; a coiled cord [Figure 20], either in its entire extent or partly; or a straight cord. A straight cord has a disadvantage in that it is more prone to disruption of blood flow.[33]

## Umbilical cord morphometry

**Figure 21 (A,B):**
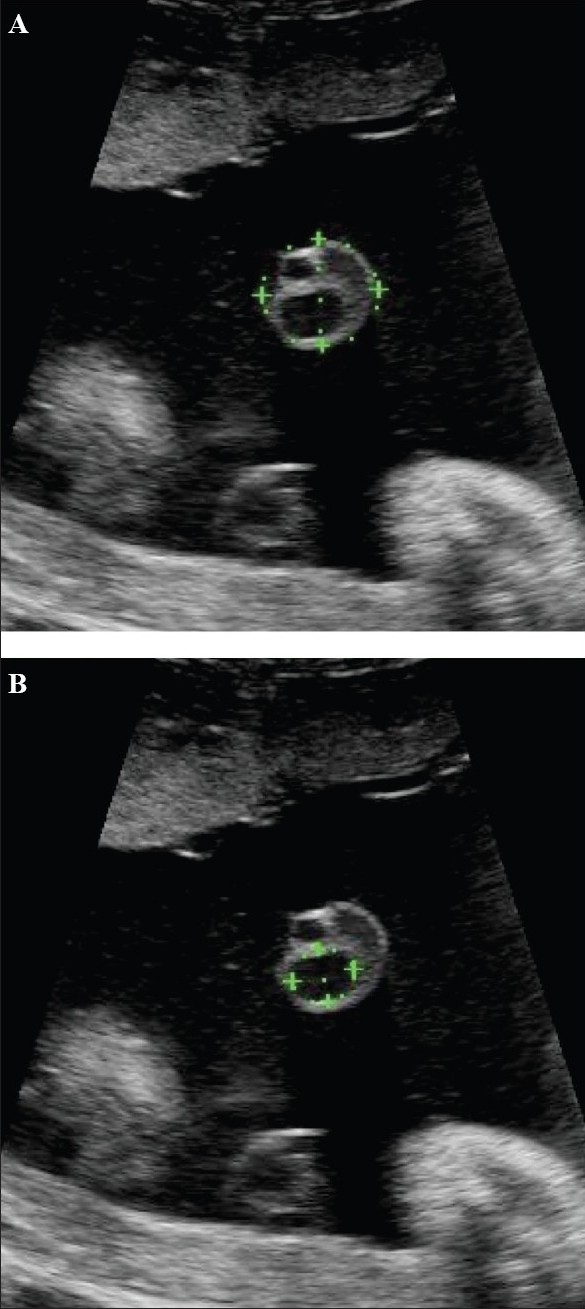
Transverse images through the cord show the method of estimation of the cross-sectional area (A) of the cord and of the umbilical vein (B)

**Figure 22:**
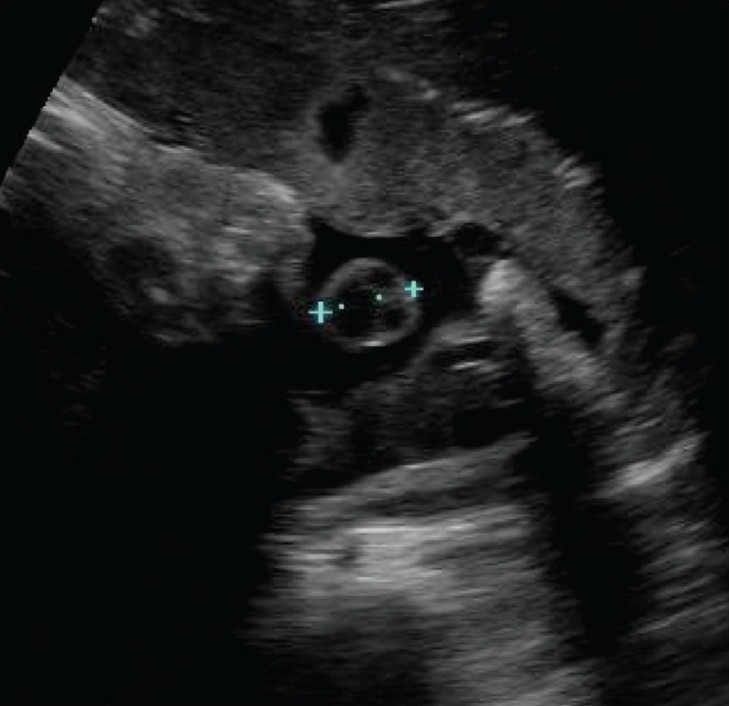
Transverse image through the cord shows the method of measurement of the maximum cord diameter

Weissman performed USG measurements of the umbilical cord, adding yet another biometric parameter to the list of measurements for a growing fetus. The amount of Wharton's jelly also could be calculated from these measurements by subtracting the area of the vein + the area of the arteries from the area of the cord [Figure 21].[34] Our nomograms of the cord (unpublished data) also follow a similar trend. The cord diameter, as well the umbilical vein and artery diameters, are easy to measure [Figure 22] and we have our own nomograms for these too (unpublished data). However, the cord area and Wharton's jelly area in our population are much lower than that reported by Weissman. The probable cause for this disparity is that the women in our groups were undernourished.

## Abnormal umbilical cord morphometry

Cord morphometry is one area that promises to enable meaningful prediction of birth outcome. Very strong associations exist between smaller cords and reduced Wharton's jelly and birth outcome. Larger cords and structural defects in Wharton's jelly are frequently seen in diabetes. Comparison of the umbilical cords of IUGR fetuses and adequate-for-gestational-age fetuses shows that cords in the former are smaller. If one considers the individual components of the cord, then it is the umbilical vein which is the better predictor of birth outcome than the artery. The cord and the Wharton's jelly area, along with the umbilical vein, form the most sensitive biometric parameters in cases of IUGR. Doppler studies have shown worsening of the umbilical artery resistance in cases of lean cords.[35] Adverse neonatal outcome and fetal distress at birth have been described by many authors. Histological findings in such cases have shown diffuse or focal abnormalities in the Wharton's jelly in the form of a reduction in amount.[36] Association between a lean cord and preeclampsia has also been described, and an interesting finding is that morphometric changes in the cord were demonstrated even in the absence of changes in the growth parameters and Doppler velocimetry.[37] Cords in patients with gestational diabetes are larger, with the larger size being attributed to an excess of Wharton's jelly.[38] We feel that in addition to the increased Wharton's jelly, the umbilical vein diameter is also more in these cases and is also a contributory factor.

## Thick umbilical cord

**Figure 23 (A,B):**
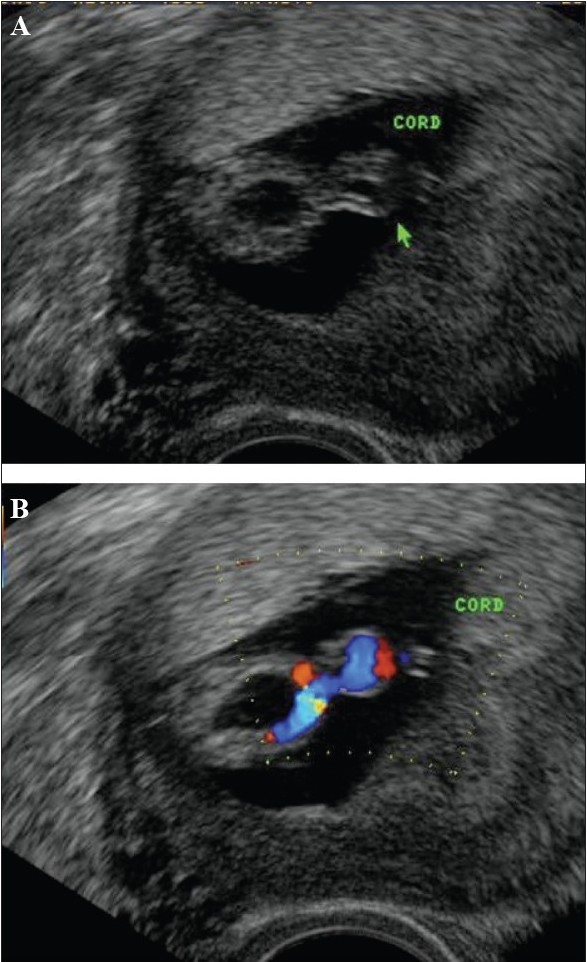
Grey-scale (A) and color Doppler (B) images of the cord suggest the presence a thick cord (arrow). The fetus had megacystis. Follow-up after a week revealed a cystic hygroma around the fetal skull; IUD occurred 1 week later

Literature describes an association between a thick umbilical cord between 10 and 14 weeks of gestation and aneuploidy [Figure 23]. A thickness of more than the 95^th^ percentile is significantly associated with aneuploidy. The thickening can persist into the second trimester. An association with an abnormal nuchal thickness (NT) and abnormal serum biochemical markers is known. It is also suggested that, like NT, the increased umbilical cord thickness can regress in the later stages of gestation.[39]

## Shorter umbilical cords in the first trimester

**Figure 24 (A, B):**
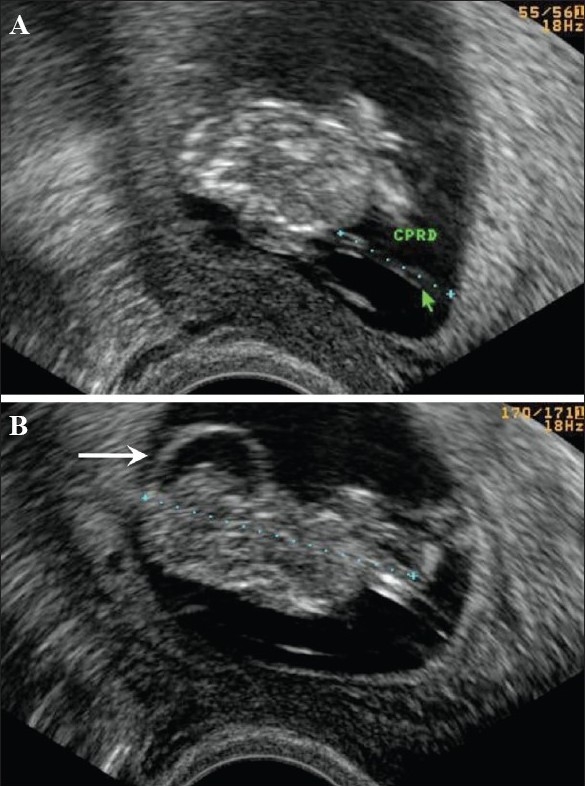
A short cord (arrow in A) is seen. The length is 1.7 cm with a CRL of 3.9 cm corresponding to a gestation of around 10 weeks and 5 days of growth. Note evidence of a cranial anomaly (arrow in B)

A cord length shorter than the CRL can predict a fetal anomaly [Figure 24]. As mentioned earlier, the CRL and the cord length are equal in the first trimester and this relationship is best evident at around 10 weeks.

Discordant size of the cord, if associated with significant difference in the CRLs in twin gestation, can also predict a poor pregnancy outcome in the smaller fetus with the thinner cord.

## Cord insertion in the first trimester

**Figure 25:**
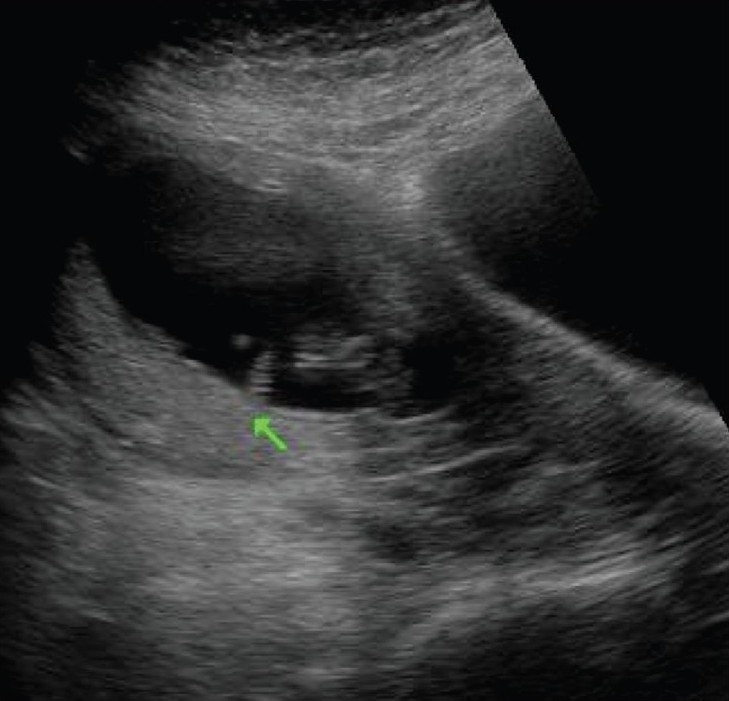
The cord insertion (arrow) in the first trimester is shown

Cord insertion in the placenta can be easily demonstrated [Figure 25]. The uterus is divided into three equal quadrants and cord insertion is assessed.[40] A low insertion of the cord can predict placental and cord abnormalities, mainly vasa previa.

## Umbilical cord cysts

**Figure 26:**
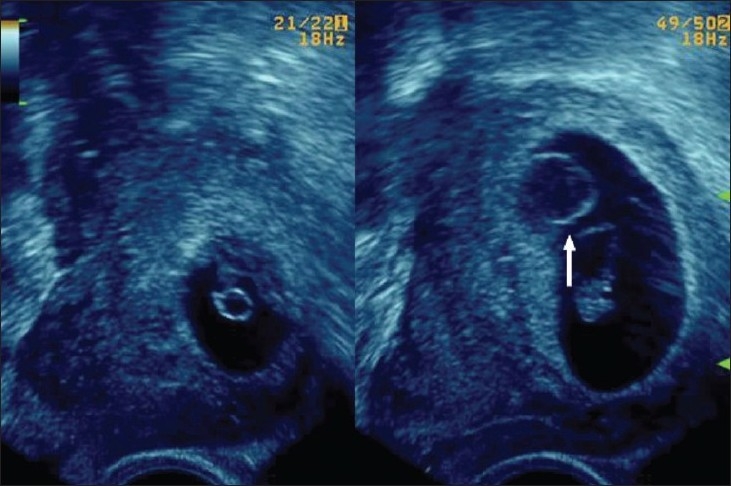
The large cystic structure (arrow) in the amniotic cavity, separate from the yolk sac, was thought to be a pseudocyst of the cord in this patient with an 8 weeks' gestation. The cyst regressed completely at 13 weeks

**Figure 27:**
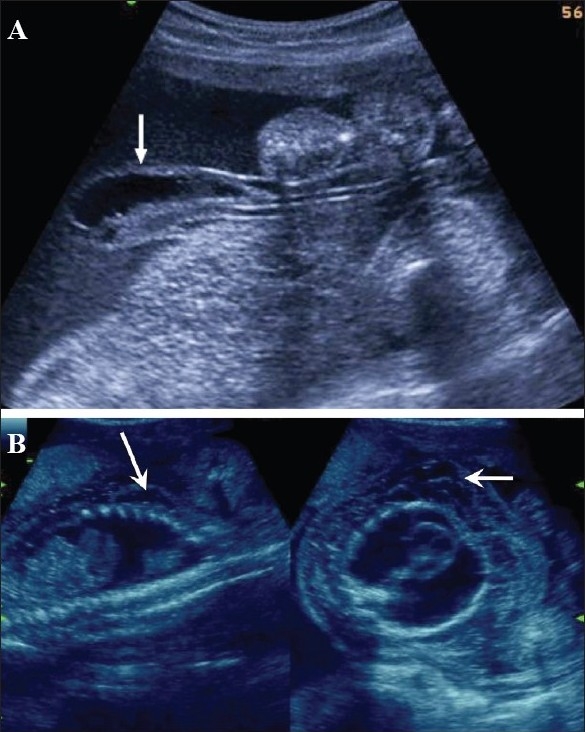
An umbilical cord cyst is seen (arrow in A) associated with hydrops (arrows in B)

**Figure 28 (A,B):**
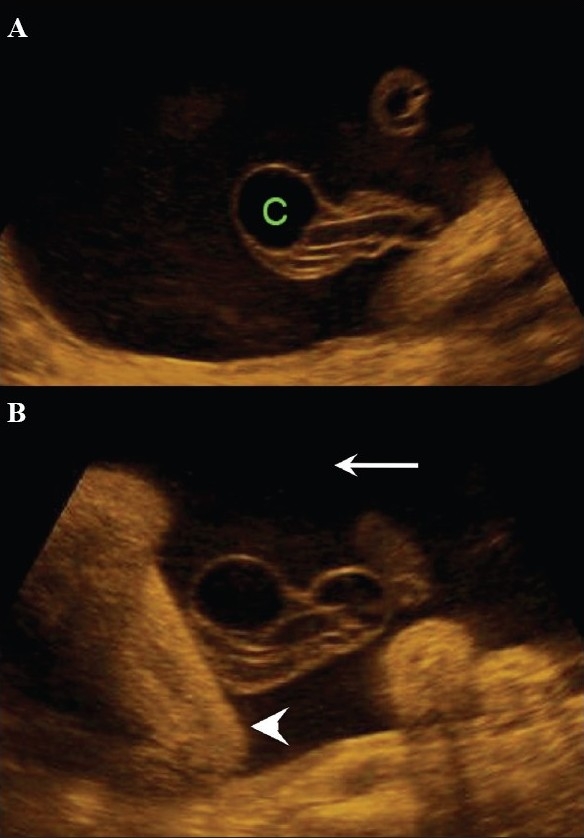
A well-defined anechoic umbilical cord cyst (c) is seen (A). Other associated findings in this case (B) include hydramnios (arrow) and arthrogryposis (arrowhead)

Umbilical cord cysts are frequently seen even in very early gestation and the majority regresses at the end of the first trimester, though some may persist [Figure 26]. Two types exist: true cysts and pseudocysts. Both have a known association with aneuploidy. The former are less common. Pseudocysts frequently regress. They have no epithelial lining and there is a localized edema of the Wharton's jelly. Umbilical cord cysts are grossly underreported. Differentiation between a true cyst and a pseudocyst is not very important since both are associated with anomalies [Figure 27 and 28]. Trisomy 13 and 18, omphaloceles, and patent urachus are the commonest associations.[41] Waldo *et al.* feel that there is a strong association of pseudocysts with chromosomal defects and structural anomalies, regardless of the USG features.[42]

**Figure 29:**
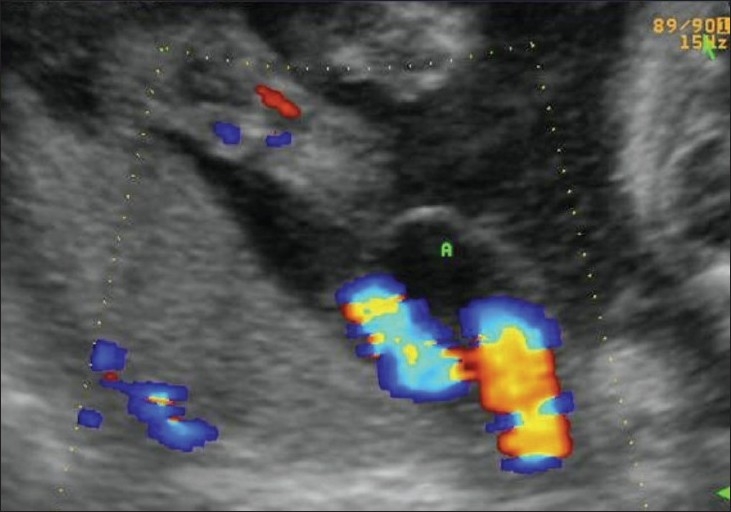
Allantoic cyst. Color Doppler helps differentiate between an allantoic cyst (A) and an umbilical cord aneurysm

Aneurysm of the umbilical artery has a poor prognosis and, if large, is known to end in fetal demise. The cause of death is, presumably, either compression of the umbilical vein or thrombosis. It is mandatory to use color Doppler to differentiate between a cyst and an aneurysm [Figure 29].[43]

Hematoma of the umbilical cord needs close monitoring in the early stages as it may be difficult to differentiate it from hemangioma of the cord. Hemangioma usually involves a longer segment of the cord and multiple hemangiomas are known to occur. It must be remembered that during the course of a vaginal delivery a hemangioma may rupture and the cord may appear completely normal after delivery. Hemangioma can be associated with chromosomal anomalies. Polyhydramnios is frequently associated with hemangioma. Fetal demise can occur in these cases due to cord compression. Other tumors, such as teratoma and angiomyxoma, are known to occur but are quite rare.[44]

## Varix of the umbilical vein

**Figure 30:**
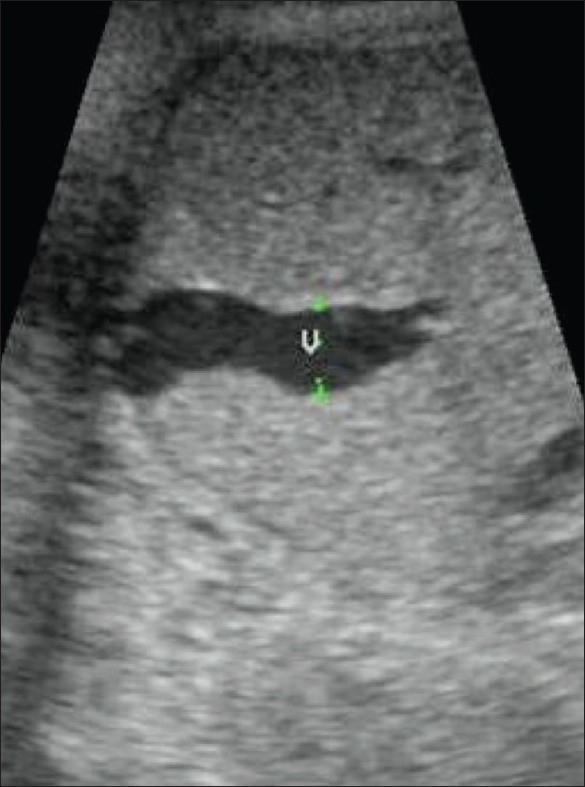
An umbilical vein varix (v) is seen, intrahepatic in location

Varix of the umbilical vein should be considered a risk factor for poor outcome. If not associated with any anomaly, the prognosis is usually good. In a reported series, the prevalence of chromosomal anomalies was 12%, while that of intrauterine death was 24%. Hydrops was the least common of the complications, with an incidence of 5%.[45] Doppler is useful in differentiating a varix from choledochal or hepatic cystic lesions.[46] Since umbilical vein varix is a rather rare entity it is not certain how it affects birth outcome, with some authors reporting a higher incidence of abnormalities and some reporting a relatively larger percentage of normal pregnancies. The message is that whenever there is evidence of an umbilical vein varix, a careful search for evidence of other anomalies and close monitoring of the pregnancy are necessary. An intrahepatic varix is more common [Figure 30].

## Anomalous course of the umbilical vein

This entity includes anomalies which can be associated with serious fetal malformations. Persistent right umbilical vein (PRUV) is the condition which heads the list of these malformations. Lack of awareness leads to the underreporting of this condition; it quite frequently occurs in isolation, without any associated fetal malformations. The following are the criteria for the diagnosis of PRUV: 1) curvature of the portal vein towards the stomach, 2) umbilical vein situated lateral to the gall bladder, and 3) umbilical vein connected to the right portal vein instead of the left. However, in the presence of other associations of the extrahepatic anomalous course, there is a high incidence of fetal anomalies and practically no system is spared.[47]

## Single umbilical artery

**Figure 31:**
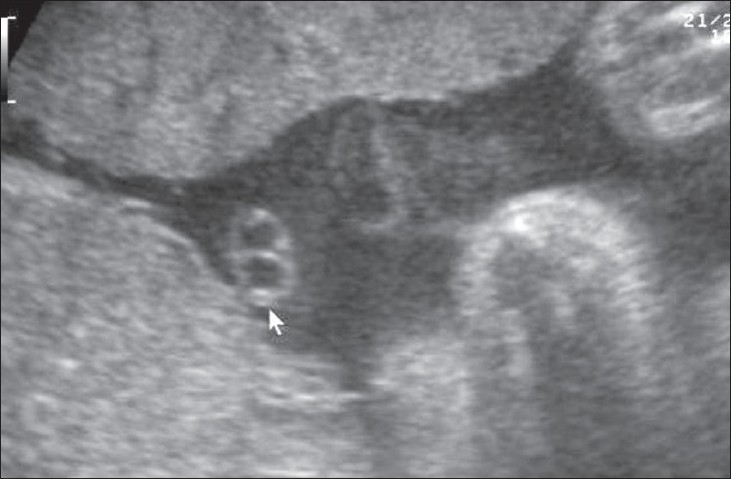
Two-vessel cord. Transverse section through the cord (arrow) shows one vein and one artery

Single umbilical artery is the most frequently encountered anomaly of the umbilical cord [Figure 31]. When it occurs in isolation, pregnancy outcome is good. However, in the presence of concurrent structural anomalies, chances of chromosomal anomalies being present are high.[48] The incidence of single umbilical artery is 0.2–1%. Probable etiologies include primary agenesis of one of the arteries, secondary atrophy of a previously normal artery, or persistence of the original single allantoic artery of the body stalk. The single umbilical artery is usually larger than normal and may be as big as a vein. The long list of associated structural anomalies spares practically no system but, amongst the chromosomal anomalies, trisomy 18, trisomy 13, and Turner syndrome are the most common.[49]

## Nuchal cord

**Figure 32:**
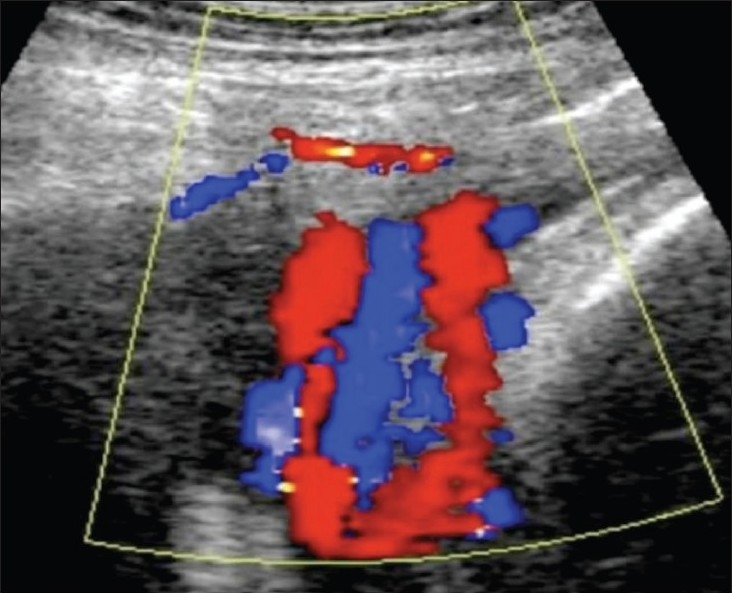
Nuchal cord. Color Dopper is excellent for demonstrating the number of loops

There cannot be a more controversial issue than the reporting of a nuchal cord. Participants in the controversy include parents, sonologists, and obstetricians. Color Doppler definitely is an advantage in the demonstration of the nuchal cord [Figure 32] but it must be remembered that a nuchal cord is not necessarily associated with any significant perinatal complication.[50] A true knot in the cord is difficult to comment upon but importance needs to be given to probable indirect evidences such as altered cardiac rhythm and absent diastolic flow in the umbilical artery.

## Amniotic fluid

There is no dispute about the importance of amniotic fluid in the assessment of fetal well-being. Estimation of the amniotic fluid volume, especially in the second and third trimesters, is a major step for the prediction of pregnancy outcome. Even before carrying out biometry and Doppler, a glance at the estimated amniotic fluid quantity can offer critical information about the gestational status. This initial impression in a case of high-risk pregnancy certainly raises the operator's confidence levels. Aberrations in the amniotic fluid volume are indicators of pregnancy-related complications. Currently, USG is the only method used in the evaluation of the amniotic fluid.

## Amniotic fluid production, resorption, and balance

A number of anatomic sites are involved in the regulation of the amniotic fluid. The volume of the amniotic fluid at any moment represents a balance between those structures that allow the passage of fluid into the amniotic cavity (such as the chorion frondosum and membranes, the skin, urinary tract, and respiratory tract) and those involved in the removal of amniotic fluid (such as the gastrointestinal tract, respiratory tract, and amniotic–chorionic interface at the uterine wall). Two additional pathways are the intramembranous and the transmembranous routes.

There are two primary sources of amniotic fluid: fetal urine and the lung liquid. There is also a minimal contribution from the fetal oronasal cavities. The two primary routes of removal are fetal swallowing and absorption of the fluid into the fetal blood. The two pathways of exchange are known as transmembranous and intramembranous; the transmembranous pathway is between the amniotic fluid and the maternal blood within the uterine wall, and the intramembranous is between the amniotic fluid and the fetal blood within the fetal surface of the placenta. The intramembranous route includes the passive exchanges which take place across the fetal skin and the umbilical cord.[51]

The amniotic cavity first appears at the blastocyst stage, 3 weeks after the last menstrual period. The chorionic cavity is formed during the fourth and the fifth menstrual weeks. By 7–8 weeks, the amniotic sac is large enough to accommodate the embryo. The amnion is visible by then on USG. The amniotic cavity completely obliterates the chorionic cavity by 12 weeks.

The amniotic fluid serves a number of important functions:

- It cushions the fetus against physical trauma.
- It allows the growth of the fetus, free from restriction or distortion by the adjacent structures.
- It provides a thermally stable environment.
- It allows the respiratory, gastrointestinal, and musculoskeletal systems to develop normally.
- It helps prevent infection.
- It provides a short-term source of fluid and nutrients to the developing embryo.

## Progress of the amniotic fluid volume

Amniotic fluid volume increases at the rate of 10 ml/week in the first 8 weeks, 25 ml/week up to 13 weeks, and 60 ml/week up to 21 weeks. After this, the weekly amniotic volume increment starts decreasing and reaches zero at around 33 weeks.

## Amniotic fluid volume measurement techniques

The three methods of assessment in use are: subjective, quantitative, and semiquantitative. Subjective evaluation is the most commonly practiced and probably shows the maximum variation in results. Experienced operators are more consistent with their results but even then, intraobserver error is not very low. The reasons for these errors include a faulty technique and absence of standardization.

The lack of standardization of the qualitative method led many researchers to devise quantitative and semiquantitative methods to describe amniotic fluid volume. The initial method for quantitative estimation of amniotic fluid volume was based on USG calculation of total intrauterine volume. This method was dependant on the use of articulated-arm static scanners. When real-time transducers replaced static scanners, this technique was not practical because the real-time transducers had a much smaller field of view and accurate measurements of the uterus were not easily obtained, particularly in late pregnancy.

## Semiquantitative method of amniotic fluid measurement

**Figure 33:**
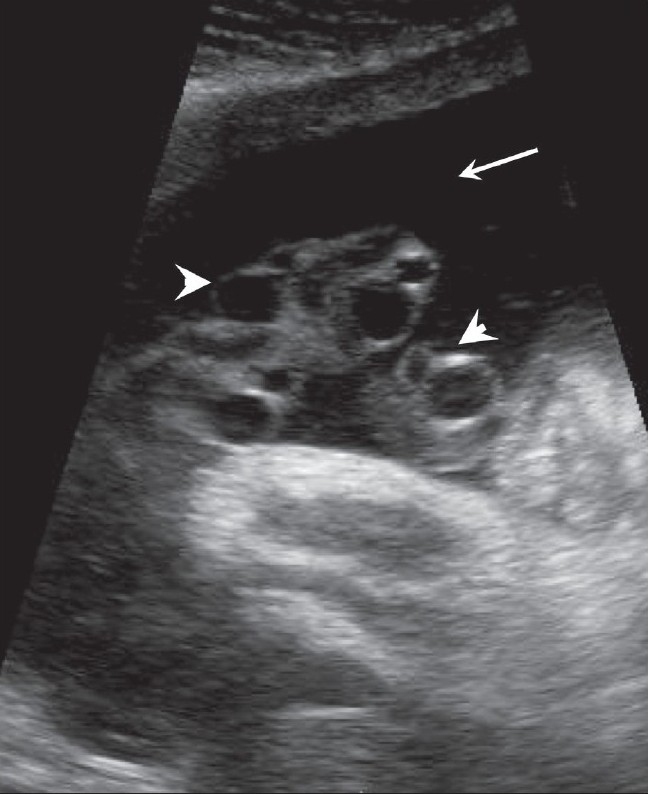
An amniotic fluid pocket (arrow) is seen containing loops of cord (arrowheads). The cord should be excluded when measuring amniotic fluid volume

Three different techniques for semiquantitative measurement of amniotic fluid have emerged. The largest vertical pocket measurement described by Chamberlain *et al.*[52] was a popular method for quite sometime. We still find it useful in the later stages of gestation. In this method, the largest vertical pocket of amniotic fluid is measured. Measurement is done at right angles to the uterine wall. Cord loops or the limbs are not included in the pocket [Figure 33]. A depth of 0–l2 cm is defined as oligohydramnios, 2.1–8 cm is considered the normal range, and a pocket of more than 8 cm is labeled as hydramnios.

The two-diameter pocket technique of Magann *et al.*[53] is based on the multiplication of the vertical depth by the horizontal diameter. The cord loops and fetal limbs are excluded. Oligohydramnios is diagnosed when the value is 0–15 cm^2^, 15.1–50 cm^2^ is considered normal, and more than 50 cm^2^ indicates hydramnios.

## Amniotic fluid index (AFI)

Phelan *et al.* proposed another method for semiquantitative assessment: calculation of amniotic fluid index. In this method, the uterus is divided into four quadrants using the linea nigra as the vertical axis and the umbilicus as the horizontal axis. The pocket with the largest vertical dimension is measured in each quadrant. The sum of the vertical measurements obtained from each of the four quadrants is the amniotic fluid index. Oligohydramnios is defined as a depth of 0–5 cm, 5.1–20 is the normal range, while hydramnios is considered when the AFI is more than 20 cm.[54] Magann compared the results of subjective operator experience and semiquantitative measurements and did not find significant differences in the results when the quantity was normal, though subjective assessment appeared to be more superior. The group thought that the two-diameter pocket measurement was the most accurate in cases of oligohydramnios, while experienced operators were the best for estimating hydramnios. In fact, even in semiquantitative methods, a subjective element still plays a major role.[55] Indian nomograms of AFI have been published.[56]

## Pitfalls in amniotic fluid estimation

**Figure 34:**
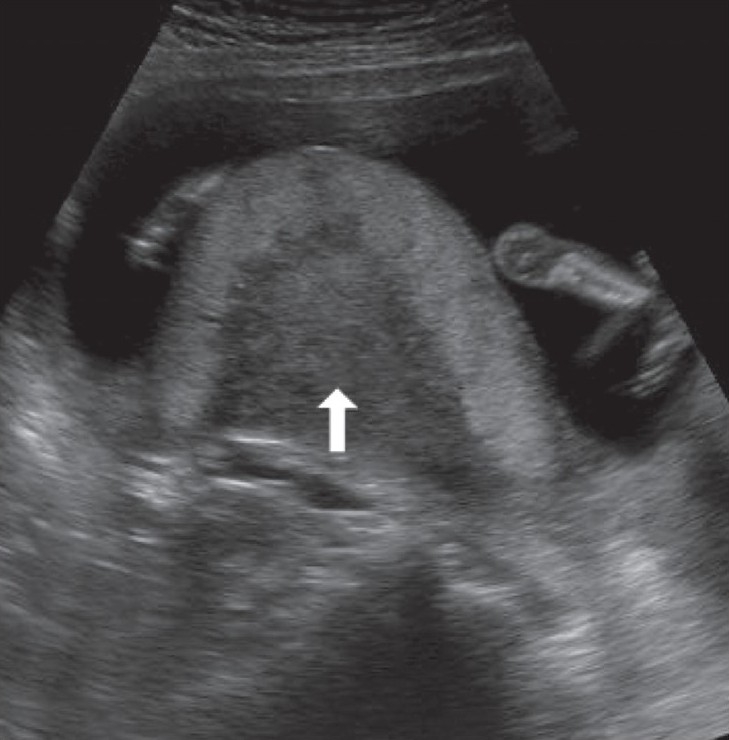
Myometrial contraction (arrow) causes underestimation of the amniotic fluid volume

The most common reason for error is excess transducer pressure.[57] Such pressure causes underestimation of the volume. Other factors include maternal obesity and floating particles in the fluid, which interfere with the visualization of the pocket. Estimation based on the measurement of a single pock*et* also has its limitations and it is not always necessary that the tallest pocket will be the correct one; for instance, its width may be less. Uterine contraction also tends to cause underestimation of the quantity of amniotic fluid [Figure 34].

## AFI and fetal weight estimation

Sriram *et al.* have stated that AFI in the late third trimester (i.e., before 38 weeks) is positively associated with fetal weight in female fetuses. The reason is probably the hormonal control in female fetuses.[58]. Prediction of fetal weight from amniotic fluid volume (AFV), however, is not a reliable or promising method.

## Oligohydramnios

Oligohydramnios can be described based on subjective as well as semiquantitative means. Subjective assessment in the second trimester relies on comparison of the fetal size and the AFV. Both occupy an almost equal volume. In the third trimester, the operator relies on the demonstration of a smaller pocket. Criteria for diagnosis by semiquantitative means have already been mentioned. Oligohydramnios is associated with structural anomalies and a poor perinatal outcome. USG assessment of this condition is crucial, as intervention is indicated in a patient without any structural anomalies and outcome can be improved.[59] Urinary tract anomalies are the commonest anomalies responsible for oligohydramnios but there are others too. Amongst the urinary tract anomalies, renal agenesis, dysplastic kidneys, autosomal recessive polycystic kidneys, and ureterovesical junction obstruction are more common. In cases of pulmonary hypoplasia, oligohydramnios is noticed much earlier in the gestation. Doppler plays an important role in the evaluation of placental insufficiency in cases of IUGR and oligohydramnios. One should bear in mind the possibility of an underlying chromosomal anomaly in the presence of oligohydramnios and a negative Doppler study.

## Polyhydramnios

**Figure 35:**
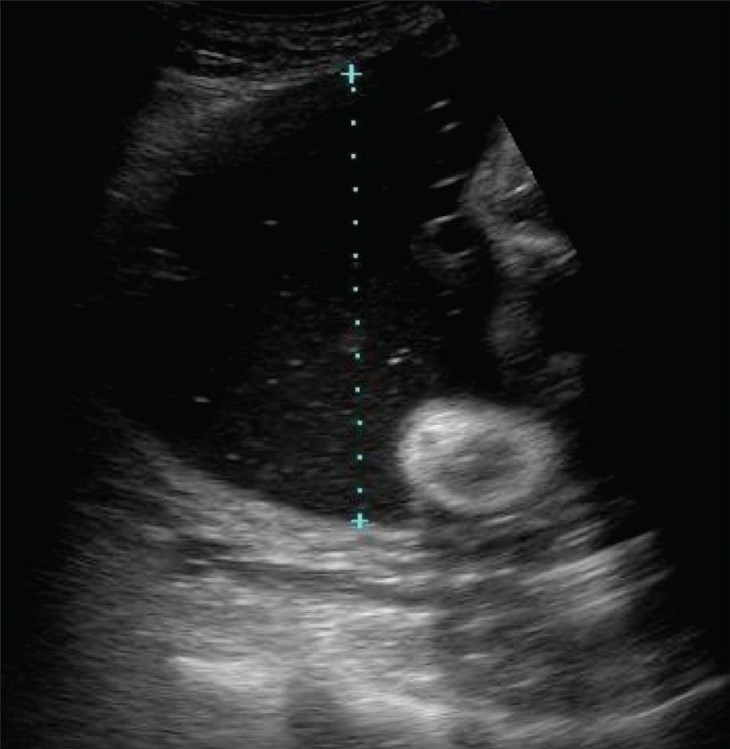
Demonstration of the technique to measure a single vertical pocket of liquor. Hydramnios is present in this case

Polyhydramnios means excess amniotic fluid; the condition is also known as hydramnios [Figure 35]. Clinical presentation is usually with a large-for-date uterus and nonpalpable fetal parts. Idiopathic hydramnios is not uncommon. Similarly, late-onset mild hydramnios is usually nonpathological. It is not unusual to come across a case of severe polyhydramnios without any demonstrable anomaly. One has to keep in mind the fact that there exist certain anomalies that may not be evident on USG. One of them is a tracheo-esophageal fistula. Sinking of the fetus at the bottom of the amniotic fluid in a case of severe hydramnios indirectly indicates a high probability of an anomaly. The long list of associated anomalies is headed by gastrointestinal tract anomalies, of which the most frequent are atresias, diaphragmatic hernias, gastroschisis, etc. Musculoskeletal anomalies too are quite common associations and so are craniospinal anomalies. Diabetes, hydrops, and twin-to-twin transfusion also are frequent contributors to the list. Preterm delivery is a common association with polyhydramnios and it is the etiology of the polyhydramnios that is more important than just the quantity of fluid. Thus the incidence of premature delivery is more common when the polyhydramnios is associated with an anomaly.[60]

## Particulate matter in amniotic fluid

**Figure 36:**
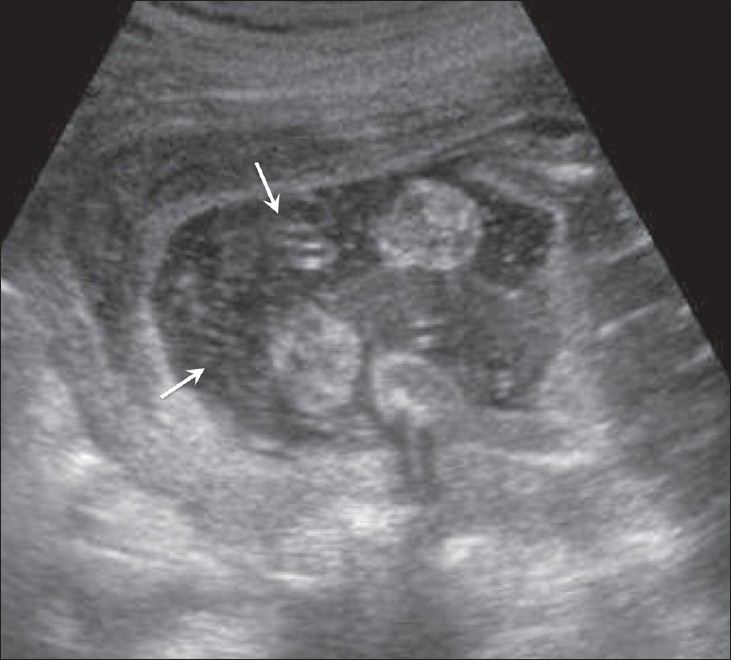
Echoes (arrows) are seen in the amniotic fluid. In this case, they were due to desquamation of the fetal skin. The postnatal diagnosis was aplasia cutis congenita. (Illustration: courtesy Dr S.T. Ambardekar)

Amniotic fluid is basically anechoic in nature. However, frequently one comes across low-level echogenic particles within the amniotic fluid. Technical factors also can alter the appearance of the amniotic fluid and increased gains or a different preset can produce this appearance in any patient. In the early years of USG, these echoes were thought to be due to vernix in the third trimester and this view holds true even today. Various researchers have reported different etiologies over the years but ultimately it was accepted that these are nonpathological. Under normal circumstances, one very often comes across this type of echogenic amniotic fluid. The differential diagnosis of these floating echogenic particles should include, apart from vernix, intraamniotic hemorrhage and infection. A recent report by Romero, attributing this appearance of echogenic sludge to infection is interesting.[61] Aplasia cutis congenita is an extremely rare dermatological disorder characterized by either localized or widespread areas of absent skin. In this condition, the bright floating internal echoes in the amniotic fluid [Figure 36] are the result of repeated epidermolysis of the fetus.[62]

## Amniotic bands

These are harmless and are thought to originate from the wrapping of chorioamniotic membranes and the uterine synechia. Amniotic band syndrome, on the other hand, is a serious associated condition which results in multiple lethal skeletal and chest deformities due to entrapment of the fetal parts.[63]

## Summary

A healthy fetal environment ensures a good birth outcome. The placenta, umbilical cord, and amniotic fluid are the main factors that decide the quality of the intrauterine environment. Any insult to the function or structure of any of these can have an impact on fetal or neonatal health. Some importance should be given to the nomograms of the placenta and the cord as they are often not looked at seriously during routine scanning. All these three contributors to the fetal environment have a strong association with chromosomal and structural defects. Thus they need careful evaluation in all stages of gestation. The fetal environment can give clues regarding future health. Its relationship with cardiovascular diseases and diabetes in adulthood is now accepted and this discovery has generated a lot of interest amongst researchers.
